# Mapping the coverage of attributes in validated instruments that evaluate primary healthcare from the patient perspective

**DOI:** 10.1186/1471-2296-13-20

**Published:** 2012-03-16

**Authors:** Jean-Frédéric Lévesque, Jeannie Haggerty, Gervais Beninguissé, Frederick Burge, David Gass, Marie-Dominique Beaulieu, Raynald Pineault, Darcy Santor, Christine Beaulieu

**Affiliations:** 1 Centre de recherche du Centre Hospitalier de l'Université de Montréal, Canada; 2 Institut national de santé publique du Québec, Montréal, Canada; 3 Agence de la santé et des services sociaux de Montréal, Montréal, Canada; 4 McGill University, Montréal, Canada; 5 Institut de formation et de recherche démographiques (IFORD), Yaoundé, Cameroun; 6 Dalhousie University, Halifax, Canada; 7 University of Ottawa, Ottawa, Canada; 8 Université de Sherbrooke, Sherbrooke, Canada

**Keywords:** Primary healthcare, Quality of healthcare, Qualitative analysis, Measurement instruments

## Abstract

**Background:**

Primary healthcare in developed countries is undergoing important reforms, and these require evaluation strategies to assess how well the population's expectations are being met. Although numerous instruments are available to evaluate primary healthcare (PHC) from the patient perspective, they do not all measure the same range of constructs. To analyze the extent to which important PHC attributes are covered in validated instruments measuring quality of care from the patient perspective.

**Method:**

We systematically identified validated instruments from the literature and by consulting experts. Using a Delphi consensus-building process, Canadian PHC experts identified and operationally defined 24 important PHC attributes. One team member mapped instrument subscales to these operational definitions; this mapping was then independently validated by members of the research team and conflicts were resolved by the PHC experts.

**Results:**

Of the 24 operational definitions, 13 were evaluated as being best measured by patients, 10 by providers, three by administrative databases and one by chart audits (some being best measured by more than one source). Our search retained 17 measurement tools containing 118 subscales. After eliminating redundancies, we mapped 13 unique measurement tools to the PHC attributes. Accessibility, relational continuity, interpersonal communication, management continuity, respectfulness and technical quality of clinical care were the attributes widely covered by available instruments. Advocacy, management of clinical information, comprehensiveness of services, cultural sensitivity, family-centred care, whole-person care and equity were poorly covered.

**Conclusions:**

Validated instruments to evaluate PHC quality from the patient perspective leave many important attributes of PHC uncovered. A complete assessment of PHC quality will require adjusting existing tools and/or developing new instruments.

## Background

Primary healthcare (PHC) in developed countries is undergoing significant changes in scope and organizational form. Depending on the reforms' main objectives and components, the evaluations may focus on different attributes, including peoples' experience of care. In addition, the notion of experience of care can be conceptualized in diverse ways and the constructs developed to measure it will vary depending on the instruments used to assess patients' perceptions and expectations. Instruments trying to capture the experience of care have looked at various attributes: access and organization of care, continuity and longitudinality, comprehensiveness, patient-centeredness and community orientation, interpersonal communication and behaviour, cultural sensitivity and discrimination, coordination and integration, empowerment and enablement, courtesy and trust.

Some instruments have divided these attributes into distinct conceptual dimensions such as organizational/structural features of care (including organizational access, visit-based continuity, integration of care, clinical team) and quality of interactions with the primary care physician (including communication, whole-person orientation, health promotion, interpersonal treatment, patient trust) [1]. Not too dissimilarly, others have grouped constructs into clinical behaviour factors and organization of care, subsuming the various attributes into different dimensions of quality related to the technical and interpersonal aspects of clinical encounters [2].

The array and overlap of attributes measured by the different instruments can generate confusion around which instruments are the most appropriate to evaluate reforms. An important first step in bringing some consistency to multiple evaluation efforts is to use a common lexicon to describe which attributes of care are covered by different instruments. The objectives of this paper are to assess the attribute coverage of various instruments that evaluate PHC from the patient perspective and to identify PHC attributes that would benefit from further instrument development. Our overall aim is to inform decision-makers and evaluation researchers about the scope of instruments available and the need for methodological developments to evaluate PHC reforms. This was part of a sequence of studies leading ultimately to in-depth, comparative psychometric information on a subset of instruments that evaluate PHC from the patient perspective, to guide decision-makers and evaluation researchers in the selection of tools to evaluate the effects of major reform initiatives in PHC.

## Method

In 2004 a Delphi consultation process with Canadian PHC experts produced operational definitions for 24 attributes that should be evaluated in current and proposed PHC models in the Canadian context [3] (Table 1). Among these, 13 were identified by the Canadian PHC experts in the study by [3], as being best measured from the patient perspective, 10 by providers, three by administrative databases and one by chart audits. This mapping builds on that study and is the groundwork for selecting a smaller subset of tools for in-depth comparative study. This study has received ethical approval from the Comité d'éthique de la recherche de l'Hôpital Charles-Lemoyne.

**Table 1:** Operational definitions of primary healthcare attributes and best data sources for their assessment

Attribute	Operational Definition	Best data source
**Clinical practice attributes dimension**		
First-contact accessibility	The ease with which a person can obtain needed care (including advice and support) from the practitioner of choice within a time frame appropriate to the urgency of the problem.	Patient
Accessibility-accommodation	The way primary healthcare resources are organized to accommodate a wide range of patients' abilities to contact healthcare clinicians and reach healthcare services.	Patient
Comprehensiveness of services	The provision, either directly or indirectly, of a full range of services to meet patients' healthcare needs. This includes health promotion, prevention, diagnosis and treatment of common conditions, referral to other clinicians, management of chronic conditions, rehabilitation, palliative care and, in some models, social services.	Patient, provider, administrative
Informational continuity Management continuity	The extent to which information about past care is used to make current care appropriate to the patient The delivery of services by different clinicians in a timely and complementary manner such that care is connected and coherent.	Patient
Technical quality of clinical care	The degree to which clinical procedures reflect current research evidence and/or meet commonly accepted standards for technical content or skill.	Provider, chart audit
**Person-oriented dimensions**		

Advocacy	The extent to which clinicians represent the best interests of individual patients and patient groups in matters of health (including broad determinants) and healthcare.	Patient
Relational continuity-	A therapeutic relationship between a patient and one or more clinicians that spans various healthcare events and results in accumulated knowledge of the patient and care consistent with the patient's needs.	Patient
Cultural sensitivity	The extent to which a clinician integrates cultural considerations into communication, assessment, diagnosis and treatment planning.	Patient
Family-centred care	The extent to which the clinician considers the family (in all its expressions) and understands its influence on a person's health and engages it as a partner in ongoing healthcare.	Patient
Interpersonal communication	The ability of the clinician to elicit and understand patient concerns, explain healthcare issues and engage in shared decision making, if desired.	Patient
Respectfulness	The extent to which health professionals and support staff meet users' expectations about interpersonal treatment, demonstrate respect for the dignity of patients and provide adequate privacy.	Patient
Whole-person care	The extent to which a clinician elicits and considers the physical, emotional and social aspects of a patient's health and considers the community context in the patient's care.	Patient
**Community-oriented dimensions**		

Client/community participation	The involvement of clients and community members in decisions regarding the structure of the practice and services provided (e.g. advisory committees, community governance).	Patient, provider
Equity	The extent to which access to healthcare and quality services are provided on the basis of health needs, without systematic differences on the basis of individual or social characteristics.	All
Intersectoral team	The extent to which the primary care clinician collaborates with practitioners from non-health sectors in providing services that influence health.	Provider
Population orientation	The extent to which the primary care clinicians assess and respond to the health needs of the population they serve.	Patient, provider
**Structural dimensions**		

Clinical information management	The adequacy of methods and systems to capture, update, retrieve, and monitor patient data in a timely, pertinent and confidential manner.	Provider
Multidisciplinary team	Practitioners from various health disciplines collaborate in providing ongoing healthcare.	Provider
Quality improvement process	The institutionalization of policies and procedures that provide feedback about structures and practices and that lead to improvements in clinical quality of care and provide assurance of safety.	Provider
System integration	The extent to which the healthcare unit organization has established and maintains linkages with other parts of the healthcare and social service system to facilitate transfer of care and coordinate concurrent care between different healthcare organizations.	Provider
**System performance**		

Accountability	The extent to which the responsibilities of professionals and governance structures are defined, their performance is monitored and appropriate information on results is made available to stakeholders.	Provider
Availability	The fit between the number and type of human and physical resources and the volume and types of care required by the catchment population served in a defined period of time.	Administrative
Efficiency/productivity	Achieving the desired results with the most cost-effective use of resources.	Administrative

### Identifying validated instruments for evaluating PHC

We searched the scientific literature for validated measurement instruments available in the public domain. We restricted our focus to instruments that evaluate primary or ambulatory care from the client or patient perspective and that address attributes or dimensions expected to change with the reforms. We first conducted a systematic electronic search of the MEDLINE and CINAHL databases using as keywords: *primary healthcare, outcome and process measurement; questionnaires; *and *psychometrics*. We eliminated questionnaires used for screening for illnesses, functional health status, or perceived outcomes of care for specific conditions (e.g. migraines, mental healthcare). We then supplemented the list by consulting local experts in health services research and by scanning references of review papers on primary care.

We identified 17 tools that had undergone different levels of quantitative and qualitative validation. Several had long and short versions (Primary Care Assessment Tool), or variations of one another (General Practice Assessment Survey and General Practice Assessment Questionnaire, both largely informed by the Primary Care Assessment Survey). After excluding these duplications, 13 unique questionnaires remained. One instrument, the Canadian Community Health Survey--Health Services Access component, was retained despite the absence of reported psychometric assessment because of its widespread use in some provincial evaluation initiatives and relevance in the Canadian context.

### Mapping the instruments' subscales to operational attributes of PHC

Information available from questionnaires was entered into linked data tables in Microsoft Access. Information on questionnaire items, subscales and psychometric properties was entered to create a measurement tools database. For each questionnaire, one of the authors (GB) matched the content of each subscale to the experts' operational definitions of PHC. A similar exercise was repeated for each individual item in the subscales. The mapping was constrained to 16 attributes that can be ascertained by patients (including 3 components of Technical Quality of Care).

To validate the mapping of subscales to attributes, we applied two different levels of rigour. We were most rigorous in mapping nine questionnaires focusing on usual source of care that were the candidate instruments for more detailed comparison in a subsequent study: the Components of Primary Care Index (CPCI, [4]); the Interpersonal Processes of Care (IPC, [5]); the EUROPEP instrument [2]; the General Practice Assessment Questionnaire (GPAQ, [6,7]); the Medical Interview Satisfaction Scale (MISS-21, [8]); the Patient Assessment of Chronic Illness Care (PACIC, [9]); the Primary Care Assessment Survey (PCAS, [1]); the Primary Care Assessment Tool (PCAT, [10,11]); and the Veterans Affairs National Outpatient Satisfaction Survey (VANOCSS, [12]). Three visit-based questionnaires (PEQ, [13]; MISS-21, [8]; COAHS, [14]) were also mapped to attributes using this method. It should be noted that the CAHPS, GPAQ and VANOCSS questionnaires also integrated a visit-based scale.

Five investigators independently examined the initial mapping. Consensus was achieved if four out of the five agreed on the original mapping or on a proposed alternate mapping. Discrepancies were resolved and final consensus was achieved in a meeting.

The remaining five questionnaires--the Consumer Assessment of Health Plans Study (CAHPS 2.0, [15]); the Canadian Community Health Survey (CCHS); the Patient Experience Questionnaire (PEQ, [13]); the Patient Satisfaction Questionnaire Short Form (PSQ-18, [16]); and the *Opinion de la clientèle au sujet des services ambulatoires *(Consumer Opinions on Ambulatory Health Services (COAHS, [14]) tool--underwent a less rigorous validation by two of the authors (JH, CB).

## Results

**Figure 1:**
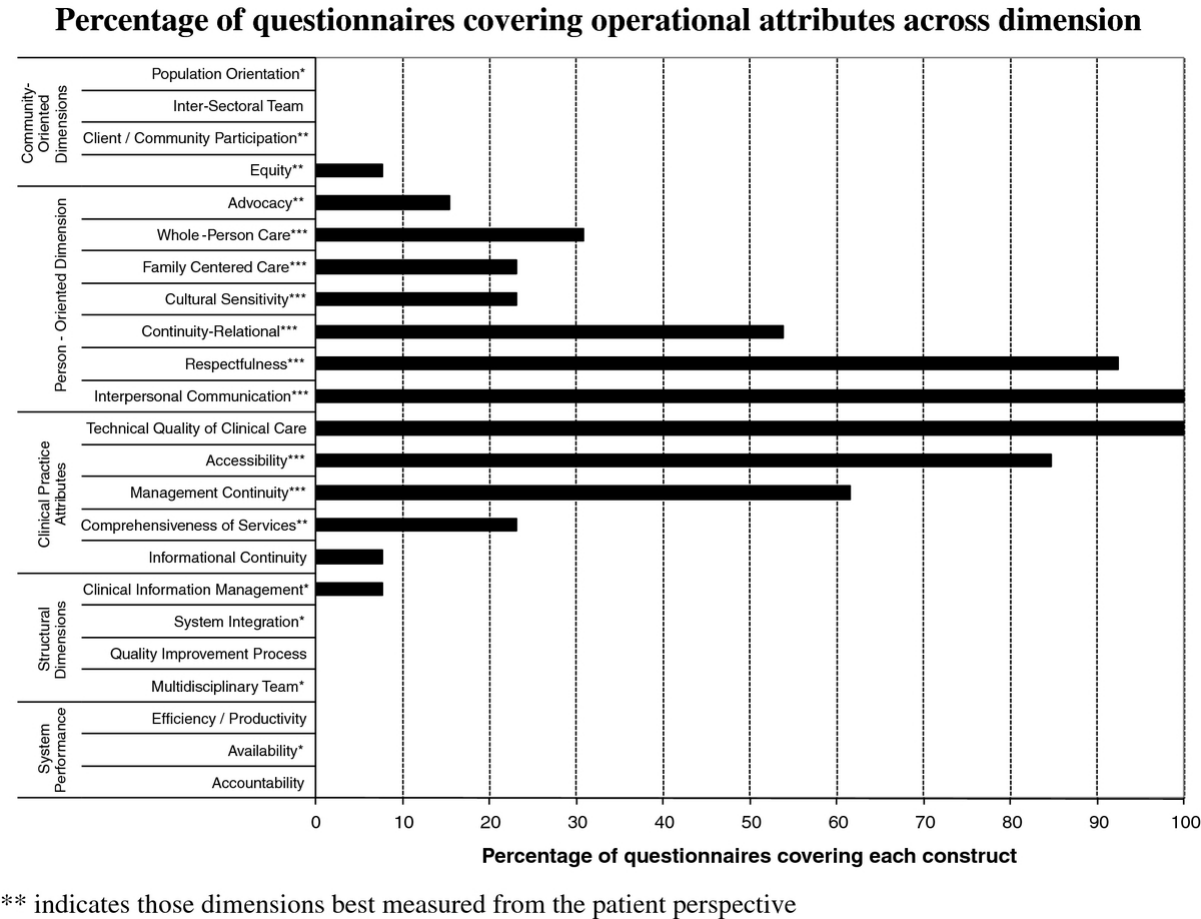
**Percentage of questionnaires covering operational attributes across dimension**.

The 17 validated questionnaires identified through the literature review comprised 118 subscales. These subscales mapped to 15 of the 24 attributes of PHC identified in our Delphi process. Nine operational attributes of PHC were not covered by any of the assessed questionnaires. Only one subscale mapped to any attributes in structural dimensions or system performance: the PCAT Coordination (Information Systems) to clinical information management. Consequently we removed the structural dimensions and system performance attributes from presentation of results. From Figure 1, we can see a high variability of coverage across attributes and that some dimensions--such as Person-Oriented and Clinical Practice Attributes Dimensions--are better covered than others. In general, the attributes best covered in terms of number of questionnaires are those that experts have evaluated as being most validly measured from the patient perspective. The community-oriented dimension has very low coverage among assessed questionnaires.

The detailed mapping of subscales to attributes, as well as items, psychometric information and developer contact are available upon request to the author. Table 2 describes the coverage of attributes by the measurement instruments. Note that both first-contact accessibility and accommodation are subsumed under accessibility and that client/community participation and intersectoral team, two attributes not measured in any instrument, are not included in the table.

**Table 2:** Coverage of operational attributes by validated instruments showing principal (•) and double (◊) mapping

Attributes	CPCI	CAHPS	IPC	EUROP	GPAQ	GPAS	CCHS	MISS-21	COAHS	PACIC	PEQ	PSQ-18	PCAS	PCAT	VANOC
Accessibility		•		•	•	•	•					•	•	•	•

Comprehensiveness of services	•													•	

Informational continuity															◊

Management continuity	•					•	•			•			•	•	•

Technical quality of clinical care			•	◊	•	•		•	•	•	•	•	•	•	

Advocacy	•												◊		

Relational continuity	•			◊	•	•							•	•	

Cultural sensitivity			•											•	

Family-centred care	•													•	

Interpersonal communication	•	•	•	•	•	•		•		•	•	•	•		•

Respectfulness		•	•	◊	•	•		•	•		•	•	•		•

Whole-person care	◊			◊										◊	

Client/community participation															

Equity			•												

Intersectoral team															

Population orientation	◊													◊	

The attributes widely covered by available instruments were accessibility, relational continuity, interpersonal communication, management continuity, respectfulness and technical quality of clinical care. In contrast, advocacy, management of clinical information, comprehensiveness of services, cultural sensitivity, family-centred care, whole-person care, population orientation and equity are poorly covered.

**Figure 2:**
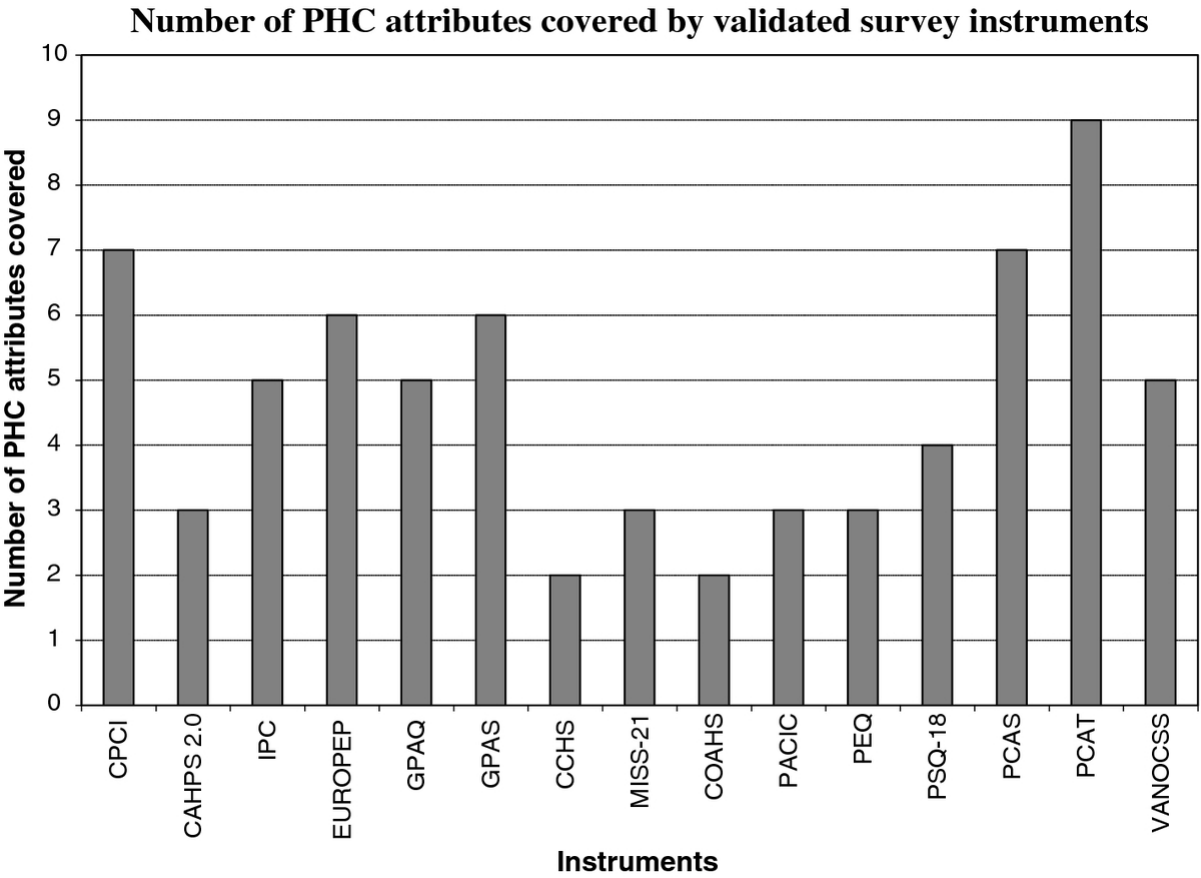
**Number of PHC attributes covered by validated survey instruments**.

Figure 2 shows the number of PHC attributes covered in each of the validated questionnaires examined in our study. Again, we see important variations, with some questionnaires covering up to nine attributes while others restrict their focus to two. The PCAT, PCAS, GPAS, CPCI, and IPC questionnaires show the best coverage, with six or more attributes covered. In contrast, the EUROPEP, CAHPS 2.0 and Canadian Community Health Survey (CCHS) show much narrower coverage, with only three attributes or fewer being covered. We also mapped individual items to attributes, and not surprisingly, found a broader coverage (results not shown), though not of attributes such as advocacy, cultural sensitivity, family-centred care, whole-person care, population orientation and equity.

## Discussion

### Assessing PHC performance from the patient perspective

In reviewing the literature, we found many tools that assess PHC attributes from the patient perspective. The 13 unique validated instruments we retained covered many of the PHC attributes identified through our previous Delphi consultation of experts [3]. However, not all aspects of PHC are covered by existing tools. Attributes related to practice structure, community orientation and system performance dimensions were scarcely addressed, while clinical practice and person-oriented attributes were widely covered. This is not surprising since more structural attributes and attributes related to communities or population aspects have been identified as not being as well measured from the patients' perspective compared to attributes related to clinical encounter and interpersonal aspects of care [3]. The clinical practice attributes were identified by the experts as core and essential to the functioning of all PHC models [17]. We can therefore conclude that although there is only partial coverage in validated instruments of all PHC attributes best addressed by patient perspective and relevant for health reform evaluation, the core attributes are well covered. To be able to get more comprehensive assessments of PHC from the patient perspective, further development is needed for items or subscales to assess advocacy, management of clinical information, comprehensiveness of services, cultural sensitivity, family-centred care, whole-person care, population orientation and equity. Some tools offer better coverage than others. The PCAT and PCAS are the two instruments with the best coverage of PHC attributes. Whether the current field of evaluative research resorts preferentially to the instruments that cover the broader range of attributes goes beyond the scope of this study but would be important to assess in future studies.

The visit-based instruments cover a narrower range of attributes than do usual care instruments. This is understandable, given that some attributes, such as those related to continuity of care or comprehensiveness; involve the notions of multiple visits or services being provided by more than one professional. Therefore, focusing on usual care enables questionnaires to touch on attributes that could be hard to evaluate in a single visit. However, some attributes related to technical quality of care or first-contact accessibility might be more accurately and precisely measured through visit-based instruments. It may therefore be relevant to integrate visit-based and usual care items in instruments to optimize the coverage of attributes addressed in PHC reforms.

### Choosing the right measurement tool for reform objectives

Our study suggests that some instruments might be more appropriate than others for specific reform evaluations. Reforms specifically targeting accessibility, for instance, would be best evaluated by instruments that provide more coverage of accessibility attributes. However, customizing tools to specific organizational reform activities remains a challenge, given that most reforms involve a complex set of measures and could produce unexpected outcomes [18-20]. Capturing this complexity and these unexpected effects requires keeping a broad scope of measurement.

Nonetheless, some specificity could be achieved by using certain instruments rather than others, according to the particular priorities addressed in various jurisdictions. This highlights the importance of clarifying the objectives of PHC evaluation before choosing an evaluative instrument. Since no single tool offers complete coverage, optimal measurement of PHC attributes may be achieved using combinations of instrument subscales. The results of the study subsequent to this one, comparing in-depth the performance of attribute measurement in six selected instrument, has now been published as a supplement [21,22]. These results can guide the selection of tools given particular evaluation objectives. However, one must keep in mind that measuring a vast array of constructs might pose challenges related to the length of the instrument to be used and the related consequences' on costs and response rates. Ultimately, the selection of instruments involves trade-offs and the instruments' coverage is only one of the aspects to be considered.

### Limitations and strengths

In this study, we limited our analyses to validated instruments available in the public domain. Therefore, although other instruments with various levels of validation or previous utilization exist as well, our results apply solely to these commonly used instruments of PHC evaluation. In addition, our mapping was independent of the instruments developers' initial intent and conceptualisation. Some instruments had a clear intent of capturing a broader array of attributes of experience of care (IPC, MMISS-21), some were specific to primary care (e.g. PCAS, PCAT) or to specific dimensions (e.g. PACIC). However, using common lexicon is strength. This study should not be seen as a summative assessment of the *quality *of the tools. Our intent was solely to map the instruments to a set of attributes being seen as important to evaluate in primary care [3].

In addition, our results are reflecting mapping of whole subscales. Mapping by items showed that some subscales cover more than one attribute. However, given that single items have much less capacity to represent constructs of interest and to measure PHC attributes validly, we believe that basing our study on subscales provided more insight into the real coverage of attributes.

We also could not assess the appropriateness of coverage of various attributes and have resorted to a simple covered/not covered dichotomy in our mapping. It would be interesting, in future research, to pay attention to the different aspects of specific attributes and to assess how the available subscales are measuring them. It could be that some tools cover more attributes than others but measure them only very superficially. Other tools that restrict their coverage may measure individual attributes in depth. However, for the purpose of evaluating PHC reforms, a balance must be struck between breadth of coverage and in-depth measurement. This study could not assess what that balance should be given the multiple contexts of PHC reform evaluation.

Our study captured the vast majority of validated tools currently available to measure experience of care from the patient perspective. We did not aim at assessing the validity of currently used non validated tools. However, many non validated tools borrow from the concepts being measured in the comprehensive set of validated tools we found. Many of these non validated tools are also very descriptive in nature, aiming at reporting facts about the experience of care more than aiming at measuring reliably an underlying construct. Therefore, we feel confident that this does not represent a major issue for our study.

We used operational definitions of attributes developed by a panel of recognized Canadian experts that represented a common understanding of PHC and its desired outcomes in the Canadian context (internal validity), but do not guarantee that the results can be generalized to other contexts (external validity). However, we could argue that trying to involve experts from various settings might have resulted in the identified attributes not being useful for the Canadian context. On the other hand, such a panel of experts would most certainly have achieved consensus on the core attributes of accessibility, continuity, comprehensiveness of services, and person-centeredness, which are the most measured. We therefore feel that our results are applicable to many contexts sharing similarities with the Canadian health system.

In addition, given that our study used published validated tools as a base of comparison, our study could not assess the coverage for more recent constructs related to patients' experience, such as self-efficacy, shared-decision making, participation and enablement. Future studies should pay attention to this both in terms of complementing the existing validated instruments with items and scales that can capture these constructs as well as in evaluating their psychometric properties.

## Conclusion

A comprehensive assessment of PHC requires measuring different attributes. The available validated instruments to evaluate the quality of PHC from the patient perspective leave many important attributes uncovered, but they do address most attributes that are considered essential. For a complete assessment of PHC quality, existing tools will need to be improved and/or new evaluation instruments developed.

## Competing interests

The authors declare that they have no competing interest.

## Authors' contributions

JFL and JH designed the study and analyses plan, conducted the analyses, drafted a first version of the manuscript and finalise the manuscript. GB realised the scoping of questionnaires and classification of items. DG, MDB, RP and DS participated in the interpretation of findings and revised earlier versions of the manuscript. CB participated in the analyses and classification of items. All authors read and approved the final manuscript.

## Pre-publication history

The pre-publication history for this paper can be accessed here:

http://www.biomedcentral.com/1471-2296/13/20/prepub
